# *Sargassum fusiforme* Fucoidan SP2 Extends the Lifespan of *Drosophila melanogaster* by Upregulating the Nrf2-Mediated Antioxidant Signaling Pathway

**DOI:** 10.1155/2019/8918914

**Published:** 2019-08-14

**Authors:** Ya Zhang, Man Xu, Chenxi Hu, Amei Liu, Junjie Chen, Chenfei Gu, Xu Zhang, Cuiping You, Haibin Tong, Mingjiang Wu, Peichao Chen

**Affiliations:** ^1^College of Life and Environmental Science, Wenzhou University, Wenzhou 325035, China; ^2^Department of Natural Resources and Environmental Studies, University of Northern British Columbia, Prince George, BC, Canada; ^3^Department of Central Laboratory, Linyi People's Hospital, Shandong University, Linyi, Shandong Province, China

## Abstract

Damage accumulated in the genome and macromolecules is largely attributed to increased oxidative damage and a lack of damage repair in a cell, and this can eventually trigger the process of aging. Alleviating the extent of oxidative damage is therefore considered as a potential way to promote longevity. SFPS, a heteropolysaccharide extracted from the brown alga *Sargassum fusiforme*, has previously been shown to alleviate oxidative damage during the aging process in mice, but whether SFPS could extend the lifespan of an organism was not demonstrated. Furthermore, the precise component within SFPS that is responsible for the antioxidant activity and the underlying mechanism of such activity was also not resolved. In this study, SP2, a fucoidan derived from SFPS, was shown to exhibit strong antioxidant activity as measured by *in vitro* radical-scavenging assays. SP2 also improved the survival rate of *D. melanogaster* subjected to oxidative stress. The flies that were fed with a diet containing SP2 from the time of eclosion displayed significant enhancement in lifespan and reduced accumulation of triglyceride at the old-age stage. In addition, SP2 markedly improved the activities of the antioxidant enzymes, superoxide dismutase (SOD), catalase (CAT), and glutathione peroxidase (GSH-Px) and reduced the contents of the malondialdehyde (MDA) and oxidized glutathione (GSSG) in old flies. Furthermore, SP2 also upregulated the expression levels of the *nuclear factor-erythroid-2-like 2* (*nfe2l2 or nrf2*) and its downstream target genes, accompanied by a dramatic reduction in the expression of *kelch-like ECH-associated protein 1* (*keap1*, a canonical inhibitor of the Nrf2) in old flies. Additional support linking the crucial role of the Nrf2/ARE pathway to the antioxidant effect of SP2 was the relatively high survival rate under heat stress for *D. melanogaster* individuals receiving SP2 supplement, an effect that was abolished by the inclusion of inhibitors specific for the Nrf2/ARE pathway. Collectively, the results indicated that SP2, a *S. fusiforme* fucoidan, could promote longevity in *D. melanogaster* by enhancing the Nrf2-mediated antioxidant signaling pathway during the aging process.

## 1. Introduction

Aging is a time-dependent and gradual decline in the physiological function of an organism, which ultimately results in diseases and death. It is now well accepted that the cause of aging and the factors that promote aging are related to genes, oxidative stress, signaling pathways, and energy metabolism [1–3]. Although the exact cause of aging is still a controversy, it is well acknowledged that these factors are interconnected and they also interact with each other. The concept that oxidative damage can promote aging is well recognized [4, 5]. Cumulative genomic and cellular damages might induce the time-dependent loss of function in living organisms, and this is predominantly a result of oxidative damage [4, 6]. However, the antiaging effect conferred by antioxidants remains inconclusive, since the extension of lifespan achieved by long-term consumption of antioxidants such as vitamin E, vitamin A, beta-carotene, and glutathione (GSH) is not consistent among different organisms [7], and in human, an increase in the mortality has even been observed [8, 9]. Therefore, the strategy of using antioxidant to promote longevity should be optimized.

Free radicals such as reactive oxygen species (ROS) and reactive nitrogen species (RNS) are considered as the major drivers of oxidative damage. These free radicals and the onset of oxidative damage can be neutralized or alleviated by detoxifying and antioxidant enzymes such as SOD, CAT, and GSH-Px, which are transcriptionally regulated by the Nrf2/ARE signaling pathway [10]. Nrf2 is a transcriptional factor that plays an indispensable role in the detoxification and antioxidant systems, and *nef2l2*, the gene encoding Nrf2, has even been proposed to act as a longevity gene [11, 12]. Unsurprisingly, knocking out the *nfe2l2* gene in mice has been shown to dramatically reduce the capacity of stress resistance and cellular protection, thus accelerating the aging process [13, 14]. Additionally, Nrf2 is highly expressed in young mice and naked mole rats [13, 15, 16]. There have been numerous studies showing that activation of the Nrf2/ARE signaling pathway can slow down the aging process or ameliorate aging-related diseases [17, 18]. However, more substantial evidence is still needed to confirm the antiaging effect conferred by the activation of the Nrf2/ARE signaling.

*Sargassum fusiforme* is a seaweed that has earned its reputation as a longevity-promoting vegetable in Northeastern Asia since it can modulate metabolism, strengthen immune response, and maintain redox homeostasis [19]. Many studies have shown that polysaccharides extracted from *S. fusiforme* possess antioxidant, antitumor, and immunomodulatory activities [19–22]. Our previous studies have revealed that SFPS, a heteropolysaccharide extracted from *S. fusiforme*, can significantly improve the antioxidant defense in aging mice by promoting Nrf2-dependent stress resistance and cellular protection [23, 24]. However, whether SFPS might also extend the lifespans of organisms, in general, remains undetermined.

In this study, we screened several fractions of polysaccharides derived from SFPS. The screening procedure combined *in vitro* cell-free antioxidant assay with *in vivo* oxidative-resistant test. The results suggested that long-term SP2 supplement could significantly prolong the lifespan of *D. melanogaster* and enhance its antioxidant capacity. Subsequent experiment further showed that the SP2 could significantly activate the CncC/Nrf2-mediated antioxidant signaling pathway, thus enhancing the stress resistance and antiaging capacity of *D. melanogaster.* Collectively, the data suggested that SP2 might promote longevity in *D. melanogaster* through activating the Nrf2/ARE signaling pathway.

## 2. Materials and Methods

### 2.1. Materials and Reagents

*Sargassum fusiforme* was harvested from the coast of Wenzhou, Zhejiang Province, China. L-Rhamnose (L-Rha), D-galacturonic acid (D-GalA), D-mannose (D-Man), and D-xylose (D-Xyl) were purchased from China National Institute for the Control of Pharmaceutical and Biological Products. Luteolin, all-trans-retinoic-acid (ATRA), D-glucuronic acid (D-GlcA), D-glucose (D-Glc), D-galactose (D-Gal), and L-arabinose (L-Ara) were purchased from Sigma-Aldrich. 1-Phenyl-3-methyl-5-pyrazolone (PMP) was purchased from Major Chemicals Co. Ltd. (Hangzhou, China). Acetonitrile (HPLC-grade) was obtained from Merck (E. Merck, Darmstadt, Germany). Other utilized chemicals used were of analytical reagent grade.

### 2.2. Extraction of Polysaccharides from *S. fusiforme*

Fresh *S. fusiforme* was dried to a constant weight and then pulverized to powder, which was then used for polysaccharide extraction as described previously [23, 24]. In brief, the powder was initially defatted in 95% ethanol, and then the heteropolysaccharide SFPS was isolated from the residual fraction by hot-water extraction and ethanol precipitation. Subsequently, fractionation of SFPS, which involved CaCl_2_ precipitation to separate the fucoidan from the alginate, yielded three fractions, designated as SP1, SP2, and SP3 (Supplementary Figure 1).

### 2.3. Stress-Induced Survival Rate Assays and Lifespan Assay

*Drosophila melanogaster* individuals (male and female) were maintained at 25°C and 60% relative humidity and under a 12 h light : 12 h dark cycle. According to the gender, the virgin flies were separated within 8 h after eclosion. They were reared on basal medium (10.5% cornmeal (*w*/*v*), 10.5% sucrose (*w*/*v*), 2.1% yeast (*w*/*v*), 1.3% agar (*w*/*v*), and 0.4% propionic acid (*v*/*v*)) without or with polysaccharide. Male flies were randomly divided into four groups, with at least 100 flies per group. One group was reared on basal medium only (Con group) while the remaining groups were each reared on basal medium plus 1.6 g/L SP1, SP2, or SP3 for 10 days. After that, the flies were transferred to a medium containing 5 mmol/L paraquat, and the number of dead flies from each group was recorded every 10 h. Lifespan assay: the lifespan assay was carried out from the time of eclosion to death. The flies were reared on basal medium without or with different concentrations of SP2 (0.4 g/L: low concentration of SP2, LSP; 0.8 g/L: median concentration of SP2, MSP; and 1.6 g/L: high concentration of SP2, HSP). The number of dead flies from each group was recorded every day until all the flies were dead. Heat stress-induced survival rate assay with/without inhibition of the Nrf2/ARE signaling pathway: to block the Nrf2/ARE signaling, flies were reared on basal medium containing, in addition to SP2, the chemical inhibitor of the Nrf2/ARE pathway, luteolin or all-trans-retinoic-acid (ATRA). The flies were divided into five groups: C or control group (no inhibitor), L1 group (15 *μ*mol/L luteolin), L2 group (30 *μ*mol/L luteolin), A1 group (0.125 g/L ATRA), and A2 group (0.25 g/L ATRA). The concentration of SP2 used was the concentration that yielded the best antiaging effect for each sex, as determined from the result of the lifespan experiment. The flies were reared in a 30°C incubator to provide heat stress, and the number of dead flies from each group was recorded every day until all the flies were dead. In addition, another lot of flies were prepared in the same way; but after 10 days, 15 flies per sample and 3 samples per group were taken for RNA extraction, whereas 25 flies per sample and 3 samples per group were used taken for protein extraction at a specific time. The RNA and protein samples were used for subsequent *q*RT-PCR and antioxidant activity assays, respectively.

### 2.4. HPLC Analysis

The molecular weight of SP2 was determined by high-performance gel-permeation chromatography (HPGPC) using a Waters HPLC apparatus equipped with a TSK-gel G-5000PWXL column (*Φ*7.8 mm × 300 mm ID, TOSOH, Japan) and a Waters 2414 refractive index detector. The SP2 sample (20 *μ*L of 5 mg/mL preparation) was injected into the column and eluted with 0.2 M NaCl at a flow rate of 0.6 mL/min and a column temperature of 45°C. The molecular weights were estimated by reference to the calibration curve obtained from the elution of Dextran T-series standards of known molecular weights. HPLC was performed with a Hypersil ODS-3 column (4.6 mm × 250 mm) using a mobile phase consisting of 83.0% (*v*/*v*) phosphate-buffered saline (PBS, 0.1 mol/L, pH 6.9) and 17% acetonitrile (*v*/*v*). The flow rate was set at 0.8 mL/min, and the eluent was monitored by absorbance at 254 nm. D-Glc, D-Gal, L-Ara, L-Rha, D-Xyl, D-Man, L-Fuc, D-GalA, and D-GlcA were used as reference monosaccharides.

### 2.5. Fourier Transform-Infrared (FT-IR) Spectroscopy

The FT-IR spectrum of the SP2 was obtained with a Tensor 27 spectrophotometer (Bruker Daltonics, Ettlingen, Germany). The SP2 sample was mixed with KBr powder and then pressed into a 1 mm thick disk, which was then used to obtain the spectrum, recorded in the frequency range of 4000–400 cm^−1^.

### 2.6. Detection of Triglyceride (TG) Content

Twenty-five flies from each experimental group were randomly taken at a specific time point to determine the content of triglyceride. Triglyceride content was determined by measuring the concentration of soluble triglyceride. The flies were first combined and homogenized in normal saline. The homogenate was centrifuged at 3000 × *g*, and the concentration of TG in the supernatant was measured using a Genzyme Triglyceride Kit purchased from Nanjing Jiancheng Incorporation (Nanjing, China).

### 2.7. Scavenging Activity of DPPH (1,1-Diphenyl-2-Picrylhydrazyl Radical 2,2-Diphenyl-1-(2,4,6-Trinitrophenyl) Hydrazyl) Radical

The effect of SP2 on free radical-scavenging activity was evaluated by measuring the level of DPPH-scavenging activity using a DPPH-scavenging assay kit purchased from Nanjing Jiancheng Incorporation (Nanjing, China). Polysaccharide solution was prepared by dissolving the polysaccharide powder in distilled water as previously described [23]. Next, the polysaccharide solution was added to DPPH reagent (in 95% ethanol) to yield a final concentration of 0.4, 0.8, or 1.6 g/L, and the reaction mixture was thoroughly mixed by shaking and then incubated in the dark for 30 min at room temperature. After that, the absorbance of the resulting solution was read at 517 nm against a blank. DPPH radical-scavenging activity was calculated using the equation: DPPH − scavenging rate (%) = [1‐(*A*_1_‐*A*_2_)/*A*_0_] × 100%, where *A*_0_ is the absorbance of the DPPH alone, *A*_1_ is the absorbance of DPPH+polysaccharide, and *A*_2_ is the absorbance of the polysaccharide only.

### 2.8. FRAP (Ferric Ion Reducing Antioxidant Power) and ABTS (2,2′-Azino-Bis(3-Ethylbenzothiazoline-6-Sulfonic Acid)) Assays

FRAP and ABTS assays were performed using assay kits purchased from Nanjing Jiancheng Incorporation (Nanjing, China) but with an additional control consisting of the sample only to account for the absorbance due entirely to the polysaccharide [25]. FRAP reagent was prepared by mixing 300 mmol/L acetate buffer (pH 3.6) with 10 mmol/L 2,4,6-tripyridyl-s-triazine (TPTZ) and 20 mmol/L FeCl_3_ in a volume ratio of 10 : 1 : 1, while ABTS radical was prepared by mixing the ABTS solution (7 mmol/L) with potassium persulfate (2.45 mmol/L).

### 2.9. Measurements of the MDA Content and the Enzymatic Activities of CAT and SOD

Twenty-five flies from each group were randomly collected at a specific time and then homogenized as one sample. After centrifugation at 3000 × *g*, the supernatant was used for MDA, CAT, and SOD assays performed with assay kits purchased from Nanjing Jiancheng Incorporation (Nanjing, China). The principles of the assay kits were based on previously described methods [26, 27].

### 2.10. *q*RT-PCR Analysis

Total RNA was extracted from whole flies using the TRIzol reagent. Fifteen flies per sample and three samples per group were taken for total RNA extraction. The total RNA extracted from each sample was then subjected to *q*RT-PCR analysis using the AffinityScript qPCR cDNA synthesis kit. The primers for the target genes were designed based on the sequences retrieved from FlyBase (http://flybase.org). The sequences of the primers are listed in Table 1. Quantitative RT-PCR assay was performed in a Roche LightCycler 480 platform using the SYBR Green II method. The transcript level of each gene was calculated by the *ΔΔ*Ct method with reference to that of the *rp49* gene and then normalized to the corresponding control group.

### 2.11. Statistical Analysis

The data were expressed as mean ± SD of, at least, three separate experiments. Statistical analyses were carried out by two-way ANOVA and Fisher's LSD multiple comparison tests by SPSS 17.0. Differences among groups were considered statistically significant at the *P* < 0.05 level.

## 3. Results

### 3.1. The Fucoidan SP2 Exhibits Excellent Antioxidant Activity

**Figure 1:**
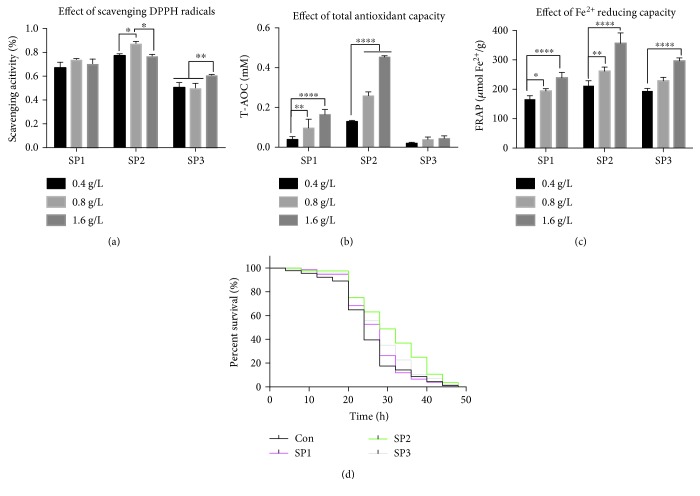
Antioxidant capacity of the different polysaccharides fractionated from SFPS. Antioxidant activity of the polysaccharides was assayed in three different cell-free systems. (a) DPPH-scavenging activity, (b) ABTS-scavenging activity, and (c) radical-scavenging activities and Fe^2+^ reducing. Data are shown as mean ± SD from three determinations. “^∗^,” “^∗∗^,” and “^∗∗∗∗^” indicate a significant difference at the *P* < 0.05, *P* < 0.01, and *P* < 0.0001 levels, respectively. (d) Fruit flies were randomly collected (100 flies/group) and reared on basal medium without (control group) or with 1.6 g/L of SP1, SP2, or SP3 for 10 days. Subsequently, the *in vivo* oxidative resistant capacity was evaluated by determining the survival rate of the flies following exposure to oxidative stress induced by 5 mmol/L paraquat, given as a diet.

It is well acknowledged that oxidative damage contributes to aging, and therefore, factors that enhance resistance to oxidative stress are considered to have antiaging effects [28–31]. We have previously reported that the SFPS, an *S. fusiforme* heteropolysaccharide, possesses antioxidant activity and shown that it can significantly alleviate aging-related stresses [23, 24]. However, the active component of SFPS responsible for the antiaging effect was not further evaluated. To address this issue, SFPS was fractionated into three fractions, which were designated as SP1, SP2, and SP3 (Supplementary Figure 1). SP1 and SP2 were both fucoidan whereas SP2 was alginate. In order to select the most promising candidate, the antioxidant capacities of SP1, SP2, and SP3 were determined by both *in vitro* and *in vivo* methods. SP2 displayed the highest antioxidant activity as shown by radical- and ion-scavenging activities (Figures 1(a)–1(c)), in particular, the ABTS clearance rate of SP2 was twice the rate of SP1 (Figure 1(b)). All three polysaccharides significantly enhanced the average survival time of *D. melanogaster* individuals that were treated with 5 mmol/L paraquat to induce oxidative stress. The flies that did not receive polysaccharide supplement died within 48 h of paraquat treatment. Twenty-four hours after paraquat treatment, the survival rate of the flies that had been given SP2 was 70%, whereas those of flies that had been given SP2 and SP3 were 55.4% and 57.2%, respectively, while flies that did not receive polysaccharide had a survival rate of just 39.5% (Figure 1(d)). The results suggested that SP2 might be an effective antioxidant, and therefore, it was chosen for further antiaging assessment.

### 3.2. Chemical Composition of SP2

The bioactivity of a compound is closely related to its chemical composition and structure. Therefore, it is vital to explore the structure-activity relationship of SP2. SP2 was determined to be a fucoidan with fucose being the major monosaccharide (52.89%), while sulfate radical, which is a unique modification of fucoidan, accounted for about 15.3% (Table 2). SP2 also contained a relatively high content of Xyl (12.1%), Gal (11.51%), and Glc (11.33%) (Table 2).

### 3.3. SP2 Promotes Longevity in *D. melanogaster*

**Figure 2:**
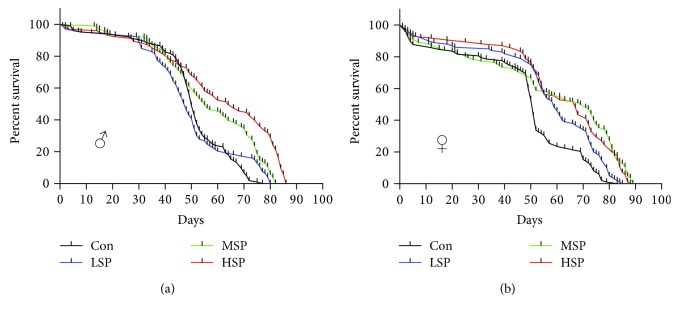
SP2 extends the lifespan of *D. melanogaster.* Male (a) and female (b) fruit flies were reared on medium containing no SP2 (Con) or different concentrations of SP2 (0.4 g/L (LSP), 0.8 g/L (MSP), and 1.6 g/L (HSP)); the number of dead flies from each group was counted daily; *n* > 250 flies.

**Figure 3 fig3:**
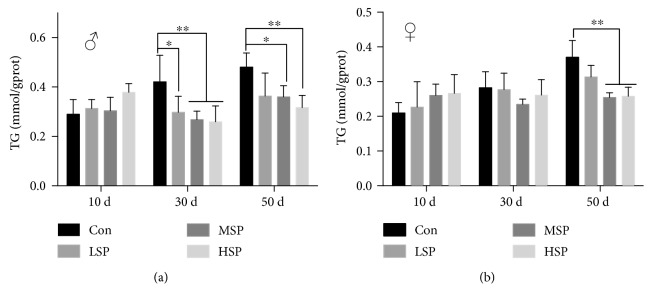
SP2 decreases the accumulation of triglyceride (TG) in *D. melanogaster* during the aging process. The flies were supplemented with varied concentrations of SP2 (0 (Con), 0.4 g/L (LSP), 0.8 g/L (MSP), and 1.6 g/L (HSP), and the contents of soluble TG in the male (a) and female (b) flies of different ages were then measured. Data are shown as the mean ± SD from three determinations, each used the extract obtained from 25 flies. “^∗^” and “^∗∗^” indicate a significant difference at the *P* < 0.05 and *P* < 0.01 levels, respectively.

The *in vitro* antioxidant activity assay and the *in vivo* oxidative stress resistance assay both demonstrated the remarkable antioxidant capacity of SP2. However, the question as to whether SP2 could promote longevity under normal condition needed to be addressed. First, the flies were reared, from the time of eclosion, on media supplemented with different concentrations of SP2. The result revealed a significant increase in lifespan for the flies receiving SP2 supplement (Figure 2, Table 3). Interestingly, the effect of SP2 on the lifespan of the flies varied with gender. At a concentration of 1.6 g/L in the medium, SP2 extended the mean lifespan by about 22%, but it extended the male lifespan by a maximum of 10%. At 0.8 g/L, SP2 extended the mean lifespan by about 24% and the female lifespan by a maximum of 10% (Table 3). Furthermore, SP2 also slowed down the accumulation of triglyceride (TG) in the aging flies (Figure 3). Old flies are known to accumulate high levels of TG because of a significant decline in their metabolism, and thus, the accumulation of TG has been considered as an aging index [32, 33]. Our data was therefore consistent with SP2 having an antiaging effect, which was clearly demonstrated by its ability to increase the lifespan of *D. melanogaster*.

### 3.4. SP2 Enhances the Endogenous Antioxidant Capacity of *D. melanogaster*

**Figure 4 fig4:**
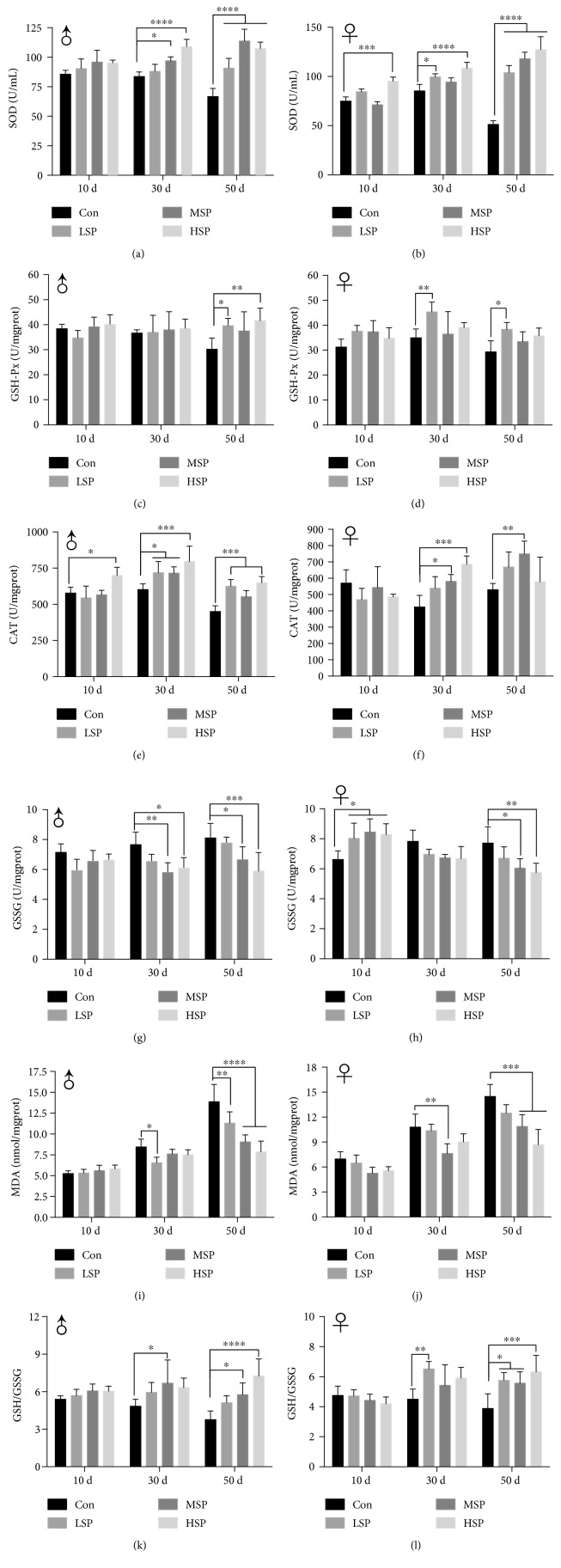
SP2-supplemented diet slows down the decline in the antioxidant capacity of *D. melanogaster* during the aging process. The fruit flies were reared on a medium containing no SP2 (Con) or different concentrations of SP2 (0.4 g/L (LSP), 0.8 g/L (MSP), and 1.6 g/L (HSP)), and their antioxidant capacity was analyzed at different ages. SOD activity (a, b); GSH-Px level (c, d); CAT activity (e, f); CSSG content (g, h); MDA content (i, j), and GSH/GSSG (k, l). The antioxidant capacity was determined for both male flies (a, d, e, g, i, and k) and female flies (b, d, f, h, j, and l). Data are shown as mean ± SD from three determinations, each used the extract obtained from 25 flies. “^∗^,” “^∗∗^,” and “^∗∗∗^” indicate a significant difference at the *P* < 0.05, *P* < 0.01, and *P* < 0.001 levels, respectively.

**Figure 5:**
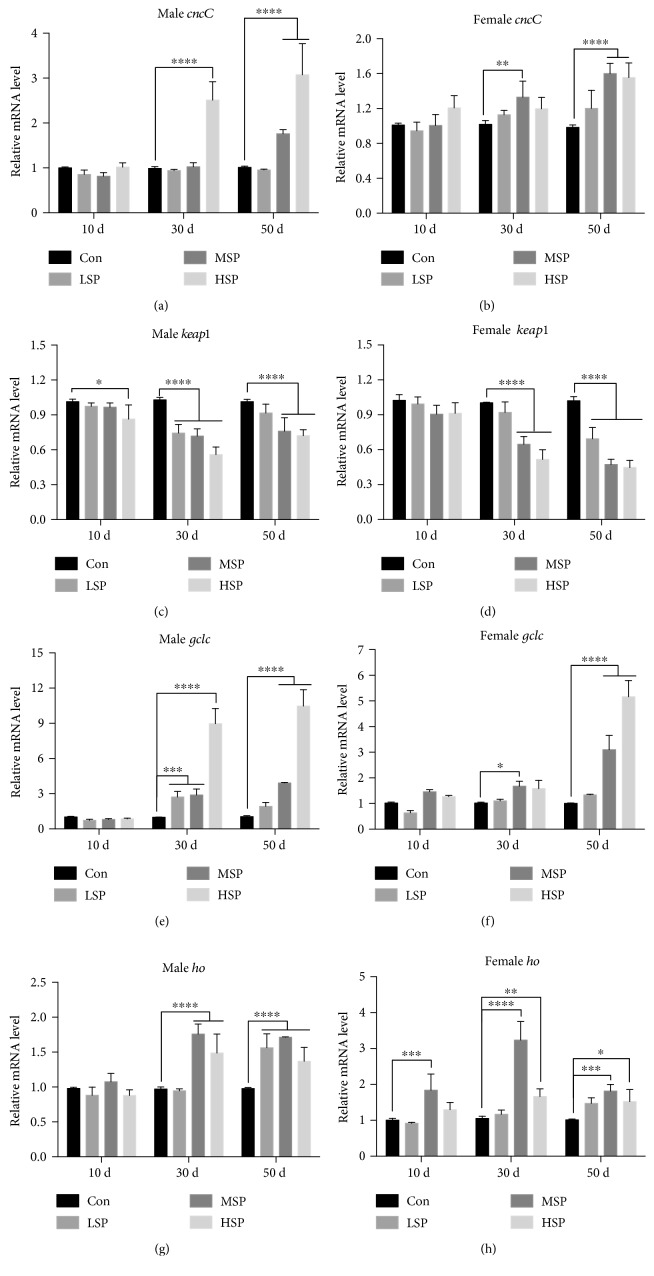
SP2 supplement upregulates the Keap1/CncC/ARE signaling pathway in aging flies. Fruit flies were reared on basal medium containing no SP2 (Con) or different concentrations of SP2 (0.4 g/L (LSP), 0.8 g/L (MSP), and 1.6 g/L (HSP)) over 50 days. The flies were taken at different time intervals, and the mRNA levels of the representative genes (*cncC, keap1*, *gclc*, and *ho*) of the Keap1/CncC/ARE signaling pathway were measured by *q*RT-PCR using the *rp49* gene as the reference gene. (a, b) *cncC*, (c, d) *keap1*, (e, f) *gclc*, and (g, h) *ho.* The expression levels of the genes were evaluated by the *ΔΔ*Ct method and normalized to those of the corresponding control. Data are shown as mean ± SD from three determinations, each used the RNA extracted from 15 flies. “^∗^,” “^∗∗^,” and “^∗∗∗^” indicate a significant difference at the *P* < 0.05, *P* < 0.01, and *P* < 0.001 levels, respectively.

Endogenous antioxidant activity is an important indicator for determining the aging status of an organism. For example, significant declines in peroxidase and superoxide dismutase activities have been observed in aging organisms, from yeast to human [34]. Analysis of the antioxidant capacity of *D. melanogaster* over time revealed an overall decrease in 50-day-old individuals (Figures 4 and 5). Furthermore, the content of MDA in these individuals also increased during the aging process, and the ratio of GSH/GSSG, commonly used as an oxidant index, was reduced. In contrast, 50-day-old flies that had been given SP2 supplement exhibited significant increases in the levels of SOD, CAT, and GSH-Px activities (Figures 4(a)–4(f)). SP2 supplement also reduced the content of MDA in these individuals by as much as 50% and increased the GSH/GSSG ratio by about two folds (Figures 4(i)–4(l)). Taken together, these results suggested that SP2 supplement could slow the decline in antioxidant defense of *D. melanogaster* during the aging process.

### 3.5. Expansion of *D. melanogaster* Lifespan by SP2 Involves the CncC/Nrf2/ARE Signaling Pathway

Nuclear factor-erythroid 2-related factor 2/antioxidant responsive element (Nrf2/ARE) is one of the most important antioxidant pathways that counteract oxidative stress and damage. Nrf2 is conserved in metazoan, and in *D. melanogaster*, it is called CncC. Previous studies have shown that SFPS can upregulate the Nrf2/ARE signaling pathway to alleviate aging stress in mice [23, 24]. Whether SP2 might stimulate the Nrf2/ARE signaling pathway to enhance the lifespan of *D. melanogaster* was the focus of this study. First, the mRNA levels of *cncC (nrf2)* and its classical inhibitor *keap1* in the flies of different ages were measured. The results revealed a remarkable upregulation of the *cncC*, and dramatic downregulation of *keap1* mRNA levels in the old flies that had been given moderate (MSP) and high (HSP) concentrations of SP2 supplement (Figures 5(a)–5(d)). The transcriptional activity of Nrf2 was further determined by measuring the mRNA levels of its representative downstream target genes: *ho* and *gclc.* As expected, the mRNA levels of these downstream antioxidant genes were markedly upregulated at the 30-day-old and 50-day-old old stages in both sexes (Figures 5(e)–5(h)). Collectively, the results suggested that the Keap1/Nrf2/ARE signaling pathway might be significantly activated by SP2 during the aging process, thereby enhancing the antioxidant capacity of *D. melanogaster* individuals that had been given SP2 supplement, with the consequence of extending their lifespans.

### 3.6. SP2-Alleviated Heat Stress Depends on the CncC/Nrf2 Signaling Pathway

**Figure 6:**
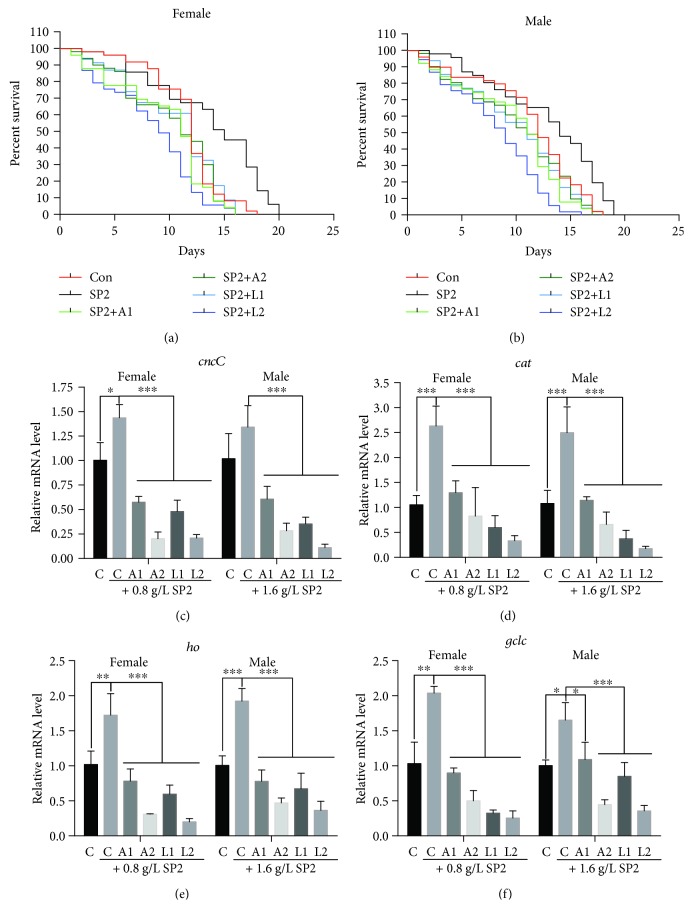
SP2-mediated stress-resistant effect in fruit flies is dependent on the CncC/Nrf2/ARE signaling. Fruit flies were reared on medium containing no SP2 (Con) or different concentrations of SP2 (0.4 g/L (LSP), 0.8 g/L (MSP), and 1.6 g/L (HSP)) without and with all-trans-retinoic-acid (A1, 0.125 g/L; A2, 0.25 g/L) or luteolin (L1, 15 *μ*mol/L; L2, 30 *μ*mol/L), and the survival rates of the flies were determined. (a) Survival rate of male flies; (b) survival rate of female flies. In addition, samples of the flies were also taken after 10 days of treatment, and the transcript levels of *cncC* (c), *cat* (d), *ho* (e), and *gclc* (f) were then measured by *q*RT-PCR using the *rp49* gene as a reference gene. The expression levels of the genes were evaluated by the *ΔΔ*Ct method, and then normalized to those of the corresponding control. Data are shown as mean ± SD from three determinations, each used the RNA extracted from 15 flies. “^∗^,” “^∗∗^,” and “^∗∗∗^” indicate a significant difference at the *P* < 0.05, *P* < 0.01, and *P* < 0.001 levels, respectively.

To validate that SP2 might exhibit antistress effect via the Nrf2-mediated signaling pathway, the Nrf2/ARE signaling was blocked by the chemical inhibitor, luteolin or ATRA [35, 36]. In order to accelerate the aging process, the flies were subjected to heat stress at 30°C. SP2 supplement significantly extended the lifespan of the flies under heat stress, but this effect was neutralized when the flies also received luteolin or ATRA in the medium (Figures 6(a) and 6(b)). Furthermore, the mRNA levels of the *cncC* gene and its representative downstream target genes, *ho*, *cat*, and *gclc*, in flies that were given just SP2 supplement were significantly upregulated compared with the control (no SP2 supplement) but declined relative to the control when the flies received both SP2 plus luteolin or ATRA (Figures 6(c)–6(f)). The effect of SP2 on stress resistance, therefore, appeared to be largely dependent on the CncC/Nrf2/ARE signaling pathway.

### 3.7. SP2 Has No Significant Effect on Body Weight

**Figure 7:**
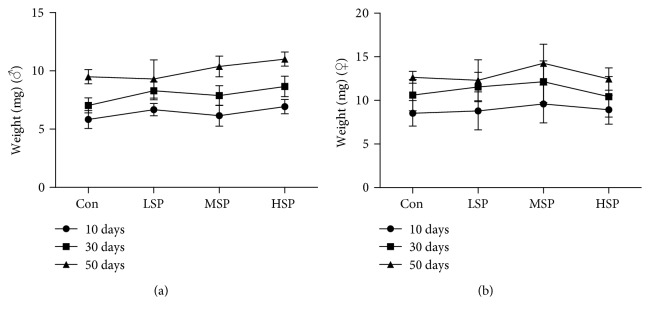
Effect of SP2 on *D. melanogaster* body weight. Body weights of male (a) and female (b) flies at different ages and reared on medium without (Con) or with different concentrations of SP2: LSP (0.4 g/L), MSP (0.8 g/L), and HSP (1.6 g/L). Data are shown as mean ± SD from three determinations, each based on 10 flies.

To investigate whether SP2 might affect food intake or trigger calorie restriction in fruit flies, the body weights of the flies given SP2 supplement were compared with those not receiving SP2. SP2 appeared to increase the body weights of the flies as measured at 10, 30, and 50 days, but the increases were not significant for both male or female groups (Figure 7). This suggested that SP2 did not restrict the food intake in *D. melanogaster*.

## 4. Discussion

The use of *Sargassum fusiforme* by Traditional Chinese Medicine to treat thyroid diseases and as a health maintenance agent was first recorded in the ancient pharmaceutical book *Shennong Bencaojing*, but information concerning the effective components and the related mechanisms is still lacking. *S. fusiforme* has even been regarded as a longevity-promoting vegetable because it helps to modulate metabolism, strengthen immune response, and maintain redox homeostasis [19]. However, the claim that it promotes longevity has not been supported by any direct evidence, despite numerous studies demonstrating that extracts prepared from *S. fusiforme*, which contain predominantly polysaccharides, have different biological activities, such as antitumor and antioxidant activities [20–22]. We have previously reported that the heteropolysaccharide SFPS extracted from *S. fusiforme* can enhance resistance to oxidative stress and even ameliorate the aging process in mice [23, 24]. However, there was no evidence to prove that *S. fusiforme* can extend the lifespan of an organism. In this study, we showed that the fucoidan SP2, which was extracted from SFPS, could markedly prolong the lifespan of *D. melanogaster*, and to the best of our knowledge, this could be the first direct evidence to support the claim that *S. fusiforme* is a longevity-promoting vegetable.

We have integrated *in vitro* and *in vivo* antioxidant assays to screen for a promising antiaging candidate from among the different fractions extracted from SFPS. Initial tests revealed SP2 to be a promising candidate, as it exhibited the best antioxidant activity and gave the highest survival rate when fed to the flies (Figure 1). SP2 was therefore chosen for further study to examine its longevity-promoting effect. Indeed, SP2 could increase the lifespan of the flies, further supporting its role as a longevity-promoting polysaccharide (Figures 2 and 3). Although the integrated screening method described in this study was robust, the extent of its validity and practicality may require further study, as the *in vitro* antioxidant assay may simply depend on a redox reaction, while the *in vivo* antioxidant assay depends on both nonenzymatic antioxidant and enzymatic systems. Previous studies that attempted to evaluate the antiaging effect of some antioxidants such as vitamin C, vitamin E, and *β*-carotene did not produce conclusive. Good consistency in antioxidant capacity between *in vivo* and *in vitro* assays was obtained for SP2 (Figure 1). Therefore, regardless of the extent of application for this antiaging screening, the data gathered for SP2 alone indicated that at least, our integrated method might provide a feasible and effective screening approach for other polysaccharides derived from brown alga, which might have the ability to slow down aging caused by oxidative damages.

It is worth noting that we merely provided one evidence of a promising antiaging compound based on its strong antioxidant activity. However, aging is a complex process that also correlates with the downregulation of metabolism and protection of cellular components against internal or external inflicted damage [37]. A decrease in antioxidant capacity and an accumulation of TG have been suggested as the hallmarks of aging [38]. The content of TG decreased significantly in 50-day-old flies but increased slightly in 10-day-old flies when the flies were given SP2 supplement (Figure 3). This suggested that SP2 significantly promoted metabolism during the aging process and yet exerted no negative impact on development in young flies. The activities of SOD, GSH-Px, and CAT and the contents of MDA and GSH are usually used to provide a comprehensive assessment of the antioxidant capacity of an organism. SP2 supplement significantly upregulated the levels of SOD, CAT, and GSH-Px activities in the aging flies but downregulated the content of MDA and the ratio of GSSG/GSH in these flies (Figure 4). Intriguingly, SP2 did not interfere with the redox balance in 10-day-old flies but comprehensively slowed down both the decline in antioxidant capacity and the increase in oxidative stress in 30-day-old and 50-day-old flies (Figure 4). In addition, the lack of statistical significance in body weight increase for the flies given SP2 supplement compared with their control counterparts suggested that long-term SP2 supplement could significantly restore the loss of antioxidant capacity in *D. melanogaster* during the aging process without having any significant effect on growth (Table 3), and this applied to both male and female flies.

Aging is also a process of time-dependent decline in function, with cumulative damage in biomacromolecules and downregulation of cellular defense and damage repair. This is reminiscent of the expression pattern of Nrf2, a crucial stress regulator in the aging process, which is downregulated in aging organisms [18, 39], although the expression starts to decrease from the middle-age stage [23]. Defect in Nrf2 can result in a decline of stress resistance and hence a shortened lifespan [13, 14]. Thus, it is of great importance to show whether reversing the decline of the Nrf2 expression would slow down the aging process. It has been validated that upregulation of the Nrf2 expression, either by genetic manipulation or by pharmacological interference, can significantly ameliorate the extent of aging-related diseases and/or retard the aging process [25, 28, 29, 40–42]. In flies given SP2 supplement, significant upregulation in the expression of *cncC* and its downstream target genes (*gclc*, *cat*, and *ho*) occurred, which further promoted stress resistance and longevity (Figures 2 [Fig fig3] [Fig fig4]–5). Conversely, the stress-resistant effect of SP2 was dramatically repressed by inhibitors of the CncC/Nrf2/ARE signaling, such as trans-retinoic-acid and luteolin (Figure 6). Although our data have clearly demonstrated the involvement of CncC/Nrf2/ARE signaling in enhancing lifespan, other anti-stress-related signaling pathways should not be excluded, since these inhibitors might also act on other genes or their products. Nevertheless, the data did indicate that the Nrf2/ARE signaling pathway plays pivotal roles in antiaging, and targeting this pathway would be a promising approach for the screening of antiaging compounds [43, 44].

However, it must be stressed that the ectopic expression of Nrf2 in an organism can adversely affect its development [45]. For example, constitutive activation of the *Nrf2* gene induces hyperkeratosis in the esophagus and forestomach, leading to postnatal lethality [46]. It is also fatal to overexpress Nrf2 during embryonic development, as Nrf2 also modulates decisions concerned with the fate of the cell [47, 48]. Therefore, spatiotemporal manipulation of Nrf2 should also be considered. Intriguingly, under normal condition, SP2 did not seem to influence the overall antioxidant capacity and CncC/Nrf2/ARE signaling pathway at the young-age stage of the flies (Figures 4 and 5), suggesting that it might have no adverse effects on postnatal development and growth. Though the underlying mechanism requires further study, this could be an important property of SP2 when carrying out future antiaging development.

An increasing number of studies suggest that polysaccharides from other sources such as mushroom, hemp seed, and okra can also upregulate the Nrf2/ARE signaling thus ameliorating oxidative damages, aging, diabetes, and other aging-related diseases [49–51]. In addition to *S. fusiform* fucoidan, the fucoidans from other algae have also been shown to enhance the Nrf2/ARE signaling, which can ameliorate liver injury and neurodegenerative diseases [52, 53]. However, the underlying mechanism by which polysaccharides upregulate the Nrf2/ARE signaling pathway remains unclear. It is possible that the bioactivities of polysaccharides may be intensively related to their molecular structures, which are determined by the molecular weight, chemical modification, monosaccharide composition, linkage types, and chain conformation of the polysaccharides. Fucoidans consist of a group of certain fucose-containing sulfated polysaccharides, which have been reported to possess many biological activities, including immunomodulatory, anti-inflammatory, antitumor, antioxidation, antivirus, and anticoagulant activities [54, 55]. However, the precise structure-activity relationships (SAR) for these polysaccharides remain largely undetermined. A fucoidan usually has a backbone of (1 → 3)-linked or (1 → 3)- and (1 → 4)-linked *α*-L-fucopyranosyl but also contains sulfated galactofucans, glucuronic acid, glucose, or xylose at different locations and to different extents [56]. Relatively high contents of xylose (12.10%), galactofucans (11.51%), and glucose substitutions (11.33%) were found in SP2 (Table 2), and this might be crucial for its antioxidant and antiaging functions. Thus, future study should focus on the relationship between monosaccharides and their bioactivity in oxidative aging.

Polysaccharides administered via diets would not be directly absorbed by the small intestine [24, 57, 58], and thus, their biological effects in the organisms are thought to be associated with their roles in modulating gut environment [59]. For this reason, we speculated that SP2 might modulate the gut environment of *D. melanogaster*, but confirmation of this aspect, including the elucidation of the relationship between microbiota composition and the structure of the SP2, will be a subject of further investigation.

## 5. Conclusion

We have optimized an effective method for screening the antiaging property of polysaccharides by integrating *in vitro* antioxidant screening and *in vivo* antioxidant resistance assay. Based on this method, SP2, a fucoidan extracted from the *S. fusiforme* heteropolysaccharide SFPS, was shown to have longevity-promoting activity. SP2 significantly activated the Nrf2/ARE signaling pathway, hence slowing down the decline in antioxidant defense capacity of *D. melanogaster* and increased its lifespan. This study has provided direct evidence of a longevity-promoting polysaccharide and revealed the worthiness of further research into SP2 as a health supplement.

## Figures and Tables

**Table 1:** The sequences of the primers.

Gene	FlyBase no.	Forward primers (5′-3′)	Reverse primers (5′-3′)
*cncC*	FBgn0262975	GTCGCCACTAAAACCGCATC	TTGTTCTTTCCACGCCGACG
*keap1*	FBgn0038475	TACGAAGATAGTGACGCCCC	GTGAAAGACGCTGGTGGAGT
*ho*	FBgn0037933	ATGACGAGGAGCAGCAGAAG	ACAAAGATTAGTGCGAGGGC
*gclc*	FBgn0040319	GAGCCATTAGTGCCGTTAGT	GTCTTTCGTCTTCGTCTTGG
*rp49*	FBgn0002626	CCCTCTTCCAGCCATCGTTC	CCACCGATCCAGACGGAGTA

**Table 2:** Chemical composition of SP2.

Molecular weight (kDa)	Sulfate content (%)	Monosaccharide composition (%)								
52.7	15.3	Man	Rha	GlcA	GalA	Glc	Gal	Xyl	Ara	Fuc
		3.50	1.77	1.11	2.61	11.33	11.51	12.10	—	52.89

Notes: Man: D-mannose; Rha: L-rhamnose; GlcA: D-glucuronic acid; GalA: D-galacturonic acid; Glc: D-glucose; Gal: D-galactose; Xyl: D-xylose; Ara: L-arabinose; Fuc: L-fucose.

**Table 3:** Effect of the SP2 to the lifespan of *D. melanogaster*.

	Male				Female			
	Con	LSP	MSP	HSP	Con	LSP	MSP	HSP
*N*	291	283	272	275	270	288	281	288
Max. lifespan	80.2 ± 1.2	84.3 ± 3.6	84.5 ± 2.2	88.4 ± 1.9^∗^	83.0 ± 1.7	85.1 ± 1.0	91.5 ± 1.1^∗^	87.1 ± 2.3
Mean lifespan	58.2 ± 2.5	56.0 ± 3.6	65.3 ± 1.9^∗^	71.1 ± 3.0^∗^	59.7 ± 1.3	66.9 ± 1.9^∗^	74.1 ± 0.9^∗^	70.4 ± 0.8^∗^

Notes: mean ± SD, ^∗^ *P* < 0.05.

## Data Availability

The data used to support the findings of this study are available from the corresponding authors upon request.

## Supplementary Materials

Supplementary Materials Figure 1: the extract technological process of the polysaccharides from *S. fusiforme*. The fresh alga was dried at 50°C and ground to powder by an electric grinder. The alga powder was defatted by 95% ethanol, and the extracts were combined and concentrated. Thereafter, isolation and purification of the heteropolysaccharides were done by centrifugation, followed by washing with 95% ethanol. The nondialyzable phase was dried by lyophilization, and the harvested polysaccharides were named the SFPS. The SFPS was resolved and fractionated into SP1, SP2, and SP3.
