# Supplementary material for: Sargassum fusiforme Fucoidan SP2 Extends the Lifespan of Drosophila melanogaster by Upregulating the Nrf2-Mediated Antioxidant Signaling Pathway
Source: Oxid Med Cell Longev. 2019 Aug 14;2019:8918914. doi: 10.1155/2019/8918914 (PMC6710776; doi:10.1155/2019/8918914)
Supplement: Supplementary Materials — Figure 1: the extract technological process of the polysaccharides from S. fusiforme. The fresh alga was dried at 50°C and ground to powder by an electric grinder. The alga powder was defatted by 95% ethanol, and the extracts were combined and concentrated. Thereafter, isolation and purification of the heteropolysaccharides were done by centrifugation, followed by washing with 95% ethanol. The nondialyzable phase was dried by lyophilization, and the harvested polysaccharides were named the SFPS. The SFPS was resolved and fractionated into SP1, SP2, and SP3. [file 8918914.f1.pdf]

Supplementary

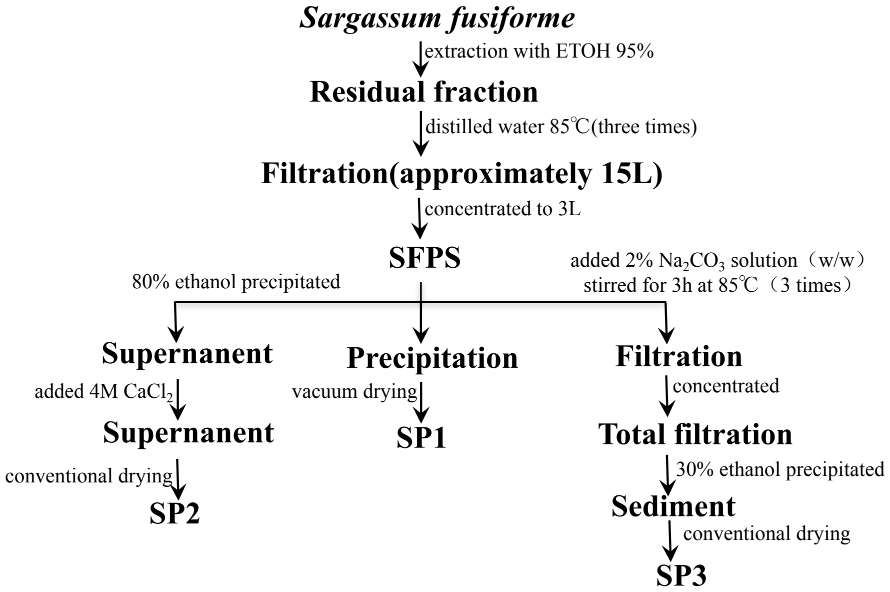

Figure 1: The extract technological process of the polysaccharides from *S. fusiforme.* The fresh alga was dried at 50 °C and ground to powder by an electric grinder. The alga powder was defatted by 95% ethanol, and the extracts were combined and concentrated. Thereafter, isolation and purification of the hetero-polysaccharides were done by centrifugation, followed by washing with 95% ethanol. The non-dialyzable phase was dried by lyophilization, and the harvested polysaccharides were named the SFPS. The SFPS was resolved and fractionated into SP1, SP2, and SP3.
